# Acute Herpes Simplex Virus Laryngitis Presenting as Airway Obstruction Post Influenza: A Viral Pneumonia

**DOI:** 10.7759/cureus.45742

**Published:** 2023-09-21

**Authors:** Tyler W Boyd, Sachin M Patil, Jason Sinclair, Dennis Chairman, Nguyen Van T, Mohammed Alnijoumi

**Affiliations:** 1 Department of Internal Medicine, Division of Pulmonary, Critical Care and Environmental Medicine, University of Missouri Health Care, Columbia, USA; 2 Department of Pathology, University of Missouri Health Care, Columbia, USA

**Keywords:** acyclovir, tracheostomy, airway obstruction, laryngitis, herpes simplex virus

## Abstract

Herpes simplex virus (HSV) typically presents with mucocutaneous or genital ulcerations but can also manifest with central nervous system involvement and occasionally other visceral or mucosal sites. However, laryngeal involvement almost exclusively presents in infants and children. Very few confirmed adult cases have been reported. Adults present with a broad spectrum of symptoms, usually in the context of significant immunocompromise. Diagnosis is difficult given a wide spectrum of nonspecific presenting symptoms and usually requires tissue biopsy. Frequently, patients have severe laryngeal edema that threatens to compromise the airway and requires tracheostomy. We present a case of HSV laryngitis in a 71-year-old female who presented with septic shock, acute renal failure, and acute hypoxic respiratory failure secondary to Influenza A and bacterial pneumonia for which she required intubation. The hospitalization course included extubation failures due to stridor, a positive cuff leak test resulting in an open tracheostomy, and a laryngeal biopsy confirming HSV infection, which was successfully treated with acyclovir.

## Introduction

Herpes simplex virus (HSV) infections typically present with mucocutaneous ulcerations. However, they can also manifest with a broader disease spectrum, including central nervous system involvement, conjunctiva, and rarely other viscera [1]. Regional or systemic HSV dissemination is known to occur among vulnerable populations, including newborn infants, patients with severe bums, malnutrition, hepatitis, and immunocompromised patients [2]. Although it was first described in 1879, laryngeal involvement is rare, especially in adults [1]. Laryngitis can be a severe, rapidly progressive manifestation of HSV that can threaten airway integrity and may require endotracheal intubation or tracheostomy. Diagnosis is difficult due to a broad spectrum of differential diagnoses with similar visual and radiographic appearances. Suspected HSV laryngitis requires a prompt biopsy, as early treatment initiation may avoid further complications. We present a case of HSV laryngitis in an immunocompetent adult admitted to the medical intensive care unit (MICU) with septic shock, acute renal failure, and acute hypoxic respiratory failure requiring intubation.

## Case presentation

A 71-year-old female patient with hypertension, hyperlipidemia, breast cancer status-post lumpectomy twice on letrozole, multiple questionable drug allergies, and chronic pain presented to an outside hospital (OSH) for acute confusion and dyspnea with hypoxia. On clinical examination, she was noted to be confused, tachypneic, hypotensive, and hypoxic requiring high flow nasal cannula up to fraction of inspired oxygen (FiO2) of 100% to keep saturation > 90%. She was intubated with a 7 mm endotracheal tube (ET), placed on ventilator support, and admitted to the OSH MICU. Clinical workup revealed leukocytosis, elevated procalcitonin, Influenza A positive, methicillin-resistant *Staphylococcus* *aureus* (MRSA) nares positive, and acute kidney injury (Table 1).

**Table 1:** Outside hospital laboratory results. MRSA: methicillin-resistant *Staphylococcus aureus*; PCR: polymerase chain reaction; COVID-19: coronavirus disease 2019; NP: nasopharyngeal; RSV: respiratory syncytialvirus;INR:International normalized ratio

TEST	RESULT	REFERENCE VALUES
Leukocytosis	17,100/mL	4000 – 11,000/mL
Platelet count	373,000/mL	150,000 – 450,000/mL
Sodium	133 mEq/L	135 – 145 mEq/L
Serum creatinine	1.51 mg/dL increased to 4.79 mg/dL at transfer	0.5 – 1 mg/dL
Lactic acid	1.8 mmol/L	< 2.2 mmol/L
Procalcitonin	3.02 ng/mL increased to 52.73 ng/mL at transfer	< 0.5 ng/mL
INR	1.12	0.9 – 1.1
Urine *Legionella* antigen	Negative	Negative
Urine streptococcal antigen	Negative	Negative
Urine analysis with microscopy	No infection	Negative for infection
Stool *Clostridium difficile* testing	Negative	Negative
MRSA nares PCR	Positive	Negative
COVID-19 NP PCR	Negative	Negative
Flu A NP PCR	Positive	Negative
Flu B NP PCR	Negative	Negative
RSV NP PCR	Negative	Negative
Urine culture	No growth	No growth
Blood culture two sets	No growth	No growth
Tracheal aspirate culture	No growth	No growth

A head computed tomography (CT) scan did not reveal any acute changes, whereas a portable chest X-ray revealed right upper lobe pneumonitis. She was started on vasopressors and hydrocortisone for acute septic shock due to superimposed bacterial pneumonia. She was treated with Tamiflu, vancomycin, and cefepime. Over the next few days, a peripherally inserted central catheter (PICC) was placed for intravenous access. Although the patient's oxygenation improved, her creatinine worsened to 4.77 mg/dL, and she was transferred to our MICU on day four after admission for acute kidney injury necessitating urgent hemodialysis.

The following day, post-arrival day one in our hospital, vasopressors were weaned, and she was initiated on hemodialysis. Labs revealed elevated creatinine, procalcitonin, C-reactive protein, and hyponatremia (Table 2).

**Table 2:** Laboratory results at our institution. TSH: thyroid stimulating hormone; ANCA: antineutrophil cytoplasmic antibody; HSV: herpes simplex virus, Ig: immunoglobulin; CMV: cytomegalovirus; MRSA: methicillin-resistant *Staphylococcus aureus*; PICC: peripherally inserted central catheter; VRE*: *vancomycin-resistant *Enterococcus faecium*

TEST	RESULT	REFERENCE VALUES
White cell count	11,870/mL	4000 – 11,000/mL
Platelet count	268,000	(150,000 – 350,000/mL)
Sodium	127	(135 -145 mEq/L)
Serum creatinine	5.25	(0.5 – 1 mg/dL)
C-reactive protein	5.36	(< 0.5 mg/dL)
Procalcitonin	31.80	(< 0.05 ng/mL)
Hemoglobin A1c	6.3%	(4% – 5.6%)
Troponin T	58	(< 14 ng/L)
TSH	0.253	(0.270 – 4.20 µunits/mL)
ANCA	Negative	Negative
Acute viral hepatitis panel	Nonreactive	Nonreactive
Respiratory pathogen panel	Influenza A positive	Negative
HSV 1 IgG	Positive	Negative
HSV 2 IgG	Negative	Negative
CMV IgG	Positive	Negative
CMV IgM	Negative	Negative
Tracheal aspirate pneumonia panel	Positive for *Acinetobacter* *baumanni*, *Enterobacter cloacae*, MRSA, Influenza A	No growth
Tracheal aspirate culture	Positive for *Acinetobacter* *baumanni*	No growth
Blood culture from PICC line	Positive for VRE *Pseudomonas putida*	No growth
Peripheral blood cultures	Negative	No growth

She completed seven days of empirical antimicrobial therapy with cefepime and vancomycin and five days of Tamiflu. A tracheal aspirate pneumonia panel returned positive for *Acinetobacter baumanni*, MRSA, *Enterobacter cloacae*, and Influenza A virus. Tracheal aspirate cultures returned positive for *A. baumanni*. Blood cultures from the peripherally inserted central catheter (PICC) grew vancomycin-resistant *Enterococcus faecium* (VRE) and *Pseudomonas putida*. Peripheral blood cultures were negative.

The PICC line was removed, and she was treated with four weeks of ampicillin-sulbactam for *A. baumanni* and two weeks of daptomycin for VRE. She received five rounds of sustained low-efficiency dialysis for volume overload before her renal function improved. Her extubation on day eight was immediately followed by stridor and a difficult reintubation with downsizing from a 7 mm to a 6 mm ET tube. She was treated with racemic epinephrine and oral prednisone 40 mg daily for five days.

**Figure 1:**
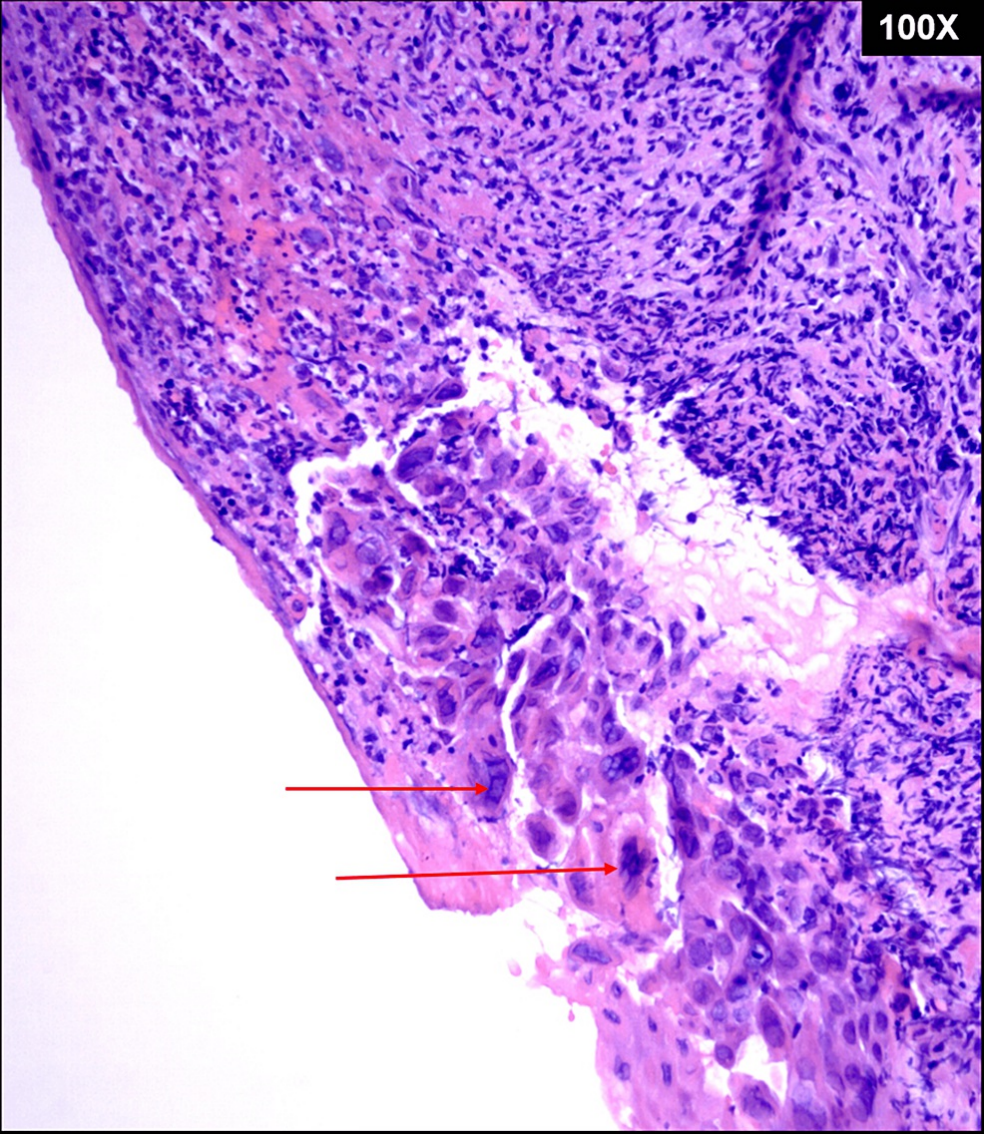
The hematoxylin and eosin-stained section of the laryngeal biopsy demonstrating the classic herpetic viral cytopathic effects. Multiple enlarged squamous cells with multinucleation (red arrows) containing multiple ground glass nuclei with chromatin margination toward the nuclear envelopes within these infected cells.

On days 15-18 of admission, the patient passed her zero-end expiratory pressure trial for mechanical ventilation weaning; however, no cuff leak was noted. The otorhinolaryngology team attempted a flexible fiberoptic laryngoscopy with poor views due to significant secretions. Due to recent intubations, there was a concern for laryngeal injury (posterior glottis versus subglottis). A soft tissue neck CT scan was recommended, which was not done due to acute kidney injury (AKI). The otorhinolaryngology team then performed an elective open tracheostomy followed by direct laryngoscopy on day 22, and post-glottic granulation tissue was noted. Laryngeal right posterior commissure biopsy revealed ulcerated mucosa with marked acute and chronic inflammation with herpetic viral cytopathic effects (Figure 1).

**Figure 2:**
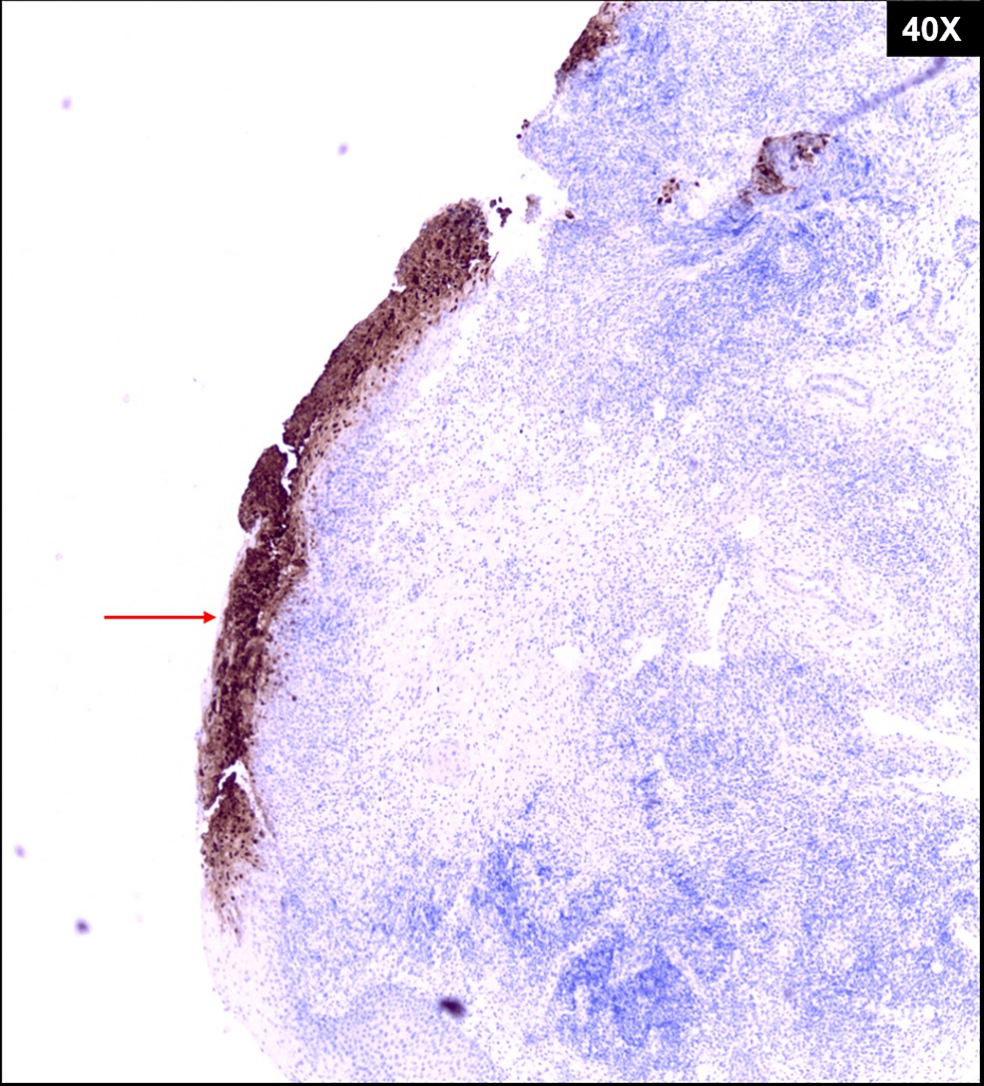
The presence of herpes simplex virus viral particles is confirmed by staining positive (red arrow) with the herpes immunohistochemical stain that was performed on the formalin fixed paraffin embedded laryngeal tissue.

Immunohistochemical stain for HSV was positive and negative for fungal infections and malignancy (Figure 2).

Viral serology was negative for active cytomegalovirus (CMV)l infection but positive for prior CMV and HSV infection (Table 2). Additional clinical history revealed no recent sexually transmitted infection, sexual encounter, or a new partner. She completed a two-week course of IV acyclovir. She was successfully transitioned to a trach collar on room air three weeks after the tracheostomy. She was transferred to long-term acute care (LTAC) facility for tracheostomy weaning.

## Discussion

Acute laryngitis is a clinical manifestation characterized by the acute onset of a hoarse voice and aphonic spells, often along with upper respiratory tract symptoms such as sore throat and nasal discharge. Although considered a self-limited disease, it has been associated with substantial morbidity among college athletes [3]. A respiratory infection frequently causes acute laryngitis in adults, and females over 36 years are affected more than males [4]. Laryngitis frequency doubles in winter when compared to summer. Respiratory viruses (rhinovirus, adenovirus, and influenza virus) are the most common cause, followed by bacteria and fungi. An increased incidence of laryngitis can be observed during the influenza epidemic [5]. HSV is a rare cause of laryngitis, with few reported proven cases in adults. Herpes viruses account for about 1% of cases of laryngitis [6]. Acute HSV laryngitis manifestation is either local or a part of disseminated HSV disease. The local disease can be due to the extension of infection from the adjoining areas or reactivation and spread via the vagus nerve [7]. Risk factors for acute HSV laryngitis include middle-aged to elderly patients (mean age of 51), immunosuppression, defective cell-mediated immunity, malnutrition, tobacco smoking, severe burns, hepatitis, inhaled corticosteroids, and superimposed respiratory infections [2,6,8]. Clinical features are variable and nonspecific, with an acute or subacute presentation, including dysphonia, dysphagia, odynophagia, dyspnea, stridor, and cough [9].

Based on the nonspecific laryngitis symptomatology, the differential diagnosis is broad, and it is difficult to rule out multiple etiologies without a tissue biopsy. For diagnosis, direct laryngoscopy with biopsy is recommended. Laryngoscopic findings include inflammatory edema of the supraglottis and glottis with or without vesicular or ulcerative lesions and vocal cord paresis [10,11]. Most adult patients presented with ulcerations followed by polypoid mass, nodular lesion, and granulation tissue [8]. Diagnosis can be established by serology, immunohistochemical (IHC) staining, or a combination of histopathology and viral culture [10]. The frequent diagnostic modality is a biopsy with IHC staining (faster), followed by viral culture with deoxyribonucleic acid detection by a polymerase chain reaction or fluorescent antibody stain [8]. Based on clinical testing data of samples from other anatomical sites, IHC staining of laryngeal biopsy is the most sensitive and specific diagnostic method [12].

Acute laryngitis symptomatic treatment includes voice rest, humidification, and pain medications [11]. Due to the significant inflammatory airway edema involving the larynx, patients can rapidly progress to airway obstruction, necessitating intubation. In some patients, tracheostomy has been done due to the concern for rapidly progressive ulceration and subglottic edema [2]. Corticosteroids and racemic epinephrine have been used as rescue agents for acute airway obstruction [2]. A retrospective review in adults concluded that corticosteroids help relieve symptoms and airway edema resolution with no adverse sequela in acute supraglottitis [13]. Antibiotics had no role as a therapeutic modality in treating typical acute laryngitis, based on a Cochrane review done in 2015. Objective outcomes were not attained, whereas subjective outcomes, such as voice hoarseness and cough, improved [14]. The Cochrane review provides insight into the etiological agents for acute laryngitis (viral > bacterial), and antibiotics had no role in viral laryngitis therapy. For acute HSV laryngitis, earlier therapy with antiviral agents (acyclovir, valacyclovir) for one to two weeks and steroids may help avoid tracheostomy [2,15]. It is prudent to consider this diagnosis earlier and utilize a laryngoscopic diagnostic biopsy for confirmation to facilitate antiviral therapy.

A systematic metareview compared HSV laryngitis in adults and pediatric patients [16]. The average age is 11 months in pediatric patients and 52 years in adults. Associated comorbid conditions in adults include malignancy, chronic obstructive pulmonary disease, diabetes mellitus, and HIV infection. Predominant symptoms in pediatric patients are stridor, dyspnea, fever, and cough; whereas, in adults, it is dysphonia, dysphagia, dyspnea, and stridor. Adults do not require airway support at a higher frequency than pediatric patients. Adults undergo tracheostomy at a higher rate, and pediatric patients have a greater rate of endotracheal intubation. Hospitalization duration is three weeks in pediatric patients and 16 days in adults. Therapeutic variances include more frequent use of intravenous corticosteroids and antivirals in the pediatric population than in adults.

Our patient was immunosuppressed due to severe acute Influenza A pneumonia with superimposed MRSA pneumonia requiring mechanical ventilatory support. The patient denied multiple sexual partners and was monogamous with her husband without oral sex. She had no active oropharyngeal or genital infections, making disseminated infection less likely. The patient probably had viral laryngitis during the second intubation when the endotracheal tube size was downgraded, which worsened once she was treated with corticosteroids leading to the absence of cuff leak during extubation trials. Also, due to airway edema and secretions, direct laryngoscopy did not provide optimal views to assess the airway. Immunosuppression in our patient probably caused reactivation of the latent HSV, spreading via the laryngeal nerves due to a change in its tropism, causing viral laryngitis [17,18]. After open tracheostomy, intraoperative direct laryngoscopy in our patient revealed significant granulation tissue within the posterior glottis region with edematous bilateral true vocal folds and a patent subglottis with no noticeable circumferential scarring. She completed the antiviral therapy and was transferred to the LTAC facility on a trach collar at room air. Superimposed HSV laryngitis can further complicate clinical management, lead to failed extubation, and significantly increase the risk of airway complications [9]. More robust outcome data is needed to understand whether earlier biopsy and treatment initiation would prevent further surgical intervention.

## Conclusions

Acute HSV laryngitis is a rare clinical presentation of HSV infection. HSV laryngitis symptoms are nonspecific, and clinical presentation is highly variable, making it a diagnostic difficulty. Laryngeal inflammation can result in acute airway obstruction requiring intubation or, as in our case, cause extubation difficulties. The diagnosis should be considered in nonresponsive acute laryngitis patients, and an early diagnostic laryngoscopy may reveal abnormal pathology. Our case report reinforces antiviral effectiveness in acute HSV laryngitis. HSV laryngitis is associated with significant morbidity, and an earlier recognition may prevent surgical interventions and complications. Even though HSV laryngitis might be rare, understanding this mechanism could have broader implications for understanding HSV and other viral infections.
